# Developmental Biology in Cyanobacteria

**DOI:** 10.3390/life9020039

**Published:** 2019-05-10

**Authors:** Antonia Herrero, Enrique Flores

**Affiliations:** Instituto de Bioquímica Vegetal y Fotosíntesis, Consejo Superior de Investigaciones Científicas, Universidad de Sevilla, 41004 Seville, Spain

Filamentous, heterocyst-forming cyanobacteria are phototrophic multicellular organisms in which N_2_-fixing heterocysts and CO_2_-fixing vegetative cells exchange regulators and nutrients. Upon nitrogen deprivation (perceived as an increase in the cellular C to N ratio), vegetative cells differentiate into heterocysts in a process that involves a specific program of gene expression and results in a heterocyst pattern consisting of two heterocysts separated by about 10 to 15 vegetative cells. In this special issue of *Life*, some aspects of heterocyst differentiation, pattern formation and biology are addressed in a collection of original research articles and reviews. In cyanobacteria, the cellular C to N ratio is sensed as levels of 2-oxoglutarate, the metabolite that lies at the link between the metabolisms of C and N assimilation. Chen et al. [1] describe a new methodology for the determination of 2-oxoglutarate levels, which is very necessary, and Shvarev et al. [2] describe two novel ABC transporter components involved in the formation of the glycolipid layer of the heterocyst envelope. Heterocyst pattern formation or maintenance is addressed in two research articles and one review. Zhang and Xu inquire about the role of some regulatory genes (hetR, hetZ, hetP) in heterocyst differentiation [3], and Fukushima and Ehira describe a novel Ser/Thr kinase necessary to keep a normal heterocyst pattern in the diazotrophic filament [4]. Additionally, Arbel-Goren et al. review a stochastic Turing model that offers a robust description of heterocyst pattern formation [5]. In turn, Flores et al. update our current knowledge on the septal junctions that mediate intercellular molecular exchange in the filament [6]. Finally, two reviews address important aspects of the biology of the heterocyst. Magnuson overviews some aspects of the bioenergetics of the heterocyst [7], and Pernil and Schleiff present a comprehensive review of heterocyst function with an emphasis on the multiple metalloproteins that function in heterocyst metabolism [8]. We expect that this collection of original articles and reviews will provide the reader with an updated view of some important aspects of heterocyst formation and function, and hence of the developmental biology of cyanobacteria. Consequently, we hope that this special issue of *Life* will capture the attention of both specialists and non-specialists who may be interested in pursuing further studies and practical applications of cyanobacteria, as well as of researchers generally interested in Bacteriology.
